# Epidemiology, Risk Factors, and Mortality in Unprovoked and Provoked Pulmonary Embolism—A Single-Center Retrospective Study in the Israeli Population: Gender and Ethnic Differences

**DOI:** 10.3390/epidemiologia7040101

**Published:** 2026-07-14

**Authors:** Raymond Farah, Nicola Luigi Bragazzi, Halil İbrahim Ceylan, Łukasz Szarpak, Agnese Maria Fioretti, Wisam Mahajna, Noor Ashqar, Rola Khamisy-Farah

**Affiliations:** 1 Internal Medicine B Department, Ziv Medical Center, Safed 13100, Israel; drraymondfa@gmail.com (R.F.); wesamahajni@hotmail.com (W.M.); noor.channel.910@gmail.com (N.A.); 2 Azrieli Faculty of Medicine, Bar Ilan University, Galilee, Safed 13100, Israel; rkhamisy@yahoo.com; 3 Laboratory for Industrial and Applied Mathematics (LIAM), Department of Mathematics and Statistics, York University, Toronto, ON M3J 1P3, Canada; 4 Department of Health Sciences (DISSAL), Postgraduate School of Public Health, University of Genoa, 16132 Genoa, Italy; 5 United Nations Educational, Scientific and Cultural Organization (UNESCO) Chair, Health Anthropology Biosphere and Healing Systems, University of Genoa, 16132 Genoa, Italy; 6 Physical Education and Sports Teaching Department, Faculty of Sports Sciences, Atatürk University, 25240 Erzurum, Turkey; halil.ibrahimceylan60@gmail.com; 7 Department of Clinical Research and Development, LUX MED Group, 02-676 Warsaw, Poland; lukasz.szarpak@gmail.com; 8 Institute of Medical Science, Collegium Medicum, The John Paul II Catholic University of Lublin, 20-950 Lublin, Poland; 9 The World Academic Council of Emergency Medicine (WACEM), Sarasota, FL 34236, USA; 10 Henry JN Taub Department of Emergency Medicine, Baylor College of Medicine, Houston, TX 77030, USA; 11 Cardio-Oncology Unit, IRCCS Istituto Tumori “Giovanni Paolo II”, 70124 Bari, Italy; amfioretti@libero.it; 12 Clalit Health Service, Akko 24124, Israel

**Keywords:** pulmonary embolism, sex/gender, ethnicity, Israel

## Abstract

Background: Pulmonary embolism (PE) is a leading cause of morbidity and mortality worldwide, ranking third among cardiovascular-related deaths after myocardial infarction and stroke. Despite extensive research, data on PE incidence and characteristics within the Israeli population remain limited. This study aimed to investigate the demographic, clinical, and prognostic factors associated with provoked (PPE) and unprovoked PE (UPE) cases in Israel. Methods: We conducted a retrospective observational study analyzing medical records of patients diagnosed with PE at Ziv Medical Center, Safed, Israel, from 2017 to 2022. Patients were classified into PPE or UPE groups based on identifiable risk factors. Demographic data, clinical characteristics, and mortality outcomes were compared using descriptive and inferential statistical methods, including the Mann–Whitney U test, chi-square test, logistic regression, Kaplan–Meier survival analysis, and Cox proportional hazards modeling. Results: A total of 348 patients (mean age: 68.6 ± 17.6 years; 54.3% female) were included, with 189 (54.3%) classified as PPE and 159 (45.7%) as UPE. Female patients were significantly older than males (*p* < 0.001), and Jewish patients were slightly older than Arab patients (*p* = 0.060). The average hospital stay was 10.7 ± 16.2 days. Although no group differences emerged in unadjusted analyses, male sex was associated with longer hospitalization and UPE with shorter hospitalization than PPE in the adjusted model. Ethnicity emerged as a significant predictor of PE type, with Jewish patients less likely to have UPE (OR = 0.457, 95% CI 0.256–0.817, *p* = 0.008). Among PPE cases, 67.2% were of Jewish origin and 32.8% were Arab, compared to 56.0% and 44.0%, respectively, in the UPE group. In-hospital mortality was 16.1% (*n* = 56). Age was a significant predictor of mortality (HR = 1.03, 95% CI 1.00–1.06, *p* = 0.020), while ethnicity, gender, and PE type showed no significant associations in multivariable models. Conclusions: Our findings highlight key demographic and clinical factors influencing PE outcomes in Israel. The significant association between ethnicity and PE type warrants further investigation to refine diagnostic and therapeutic strategies for high-risk populations.

## 1. Introduction

Pulmonary embolism (PE) represents a common and potentially life-threatening condition affecting both hospitalized and non-hospitalized patients, associated with frequent recurrences [1,2]. Beyond its acute mortality risk, PE may lead to severe long-term complications if left untreated, underscoring the importance of timely diagnosis and management. Along with deep vein thrombosis (DVT) [3], it forms the spectrum of venous thromboembolism (VTE) [4].

PE has an estimated annual incidence of 39 to 115 cases per 100,000 people, making it the third leading cause of cardiovascular death after myocardial infarction and cerebrovascular accidents, such as cerebral stroke [5,6]. Additionally, it ranks as the third most common cause of hospital-related mortality and remains the most preventable fatal complication associated with hospitalization [6].

PE occurs when a blood clot (embolus) occludes a pulmonary artery, typically originating from a thrombus in the deep venous system of the lower extremities, known as DVT. This pulmonary bed obstruction impairs gas exchange and circulation, creating a ventilation-perfusion mismatch and resulting in hypoxemia [7]. The primary risk factors for PE are encapsulated in Virchow’s triad [8,9]—hypercoagulability, venous stasis, and endothelial injury—which can be inherited or acquired. Based on these factors, PE is classified into two main categories: provoked PE (PPE) and unprovoked PE (UPE) [10,11,12].

PPE occurs in the presence of identifiable, often acquired, risk factors. These may be transient or persistent and include a personal history of VTE, active malignancy, heart or respiratory failure, coagulation disorders, hormone replacement therapy, oral contraceptive use, immobilization, recent surgery, or trauma within the past three months [11,12]. Among the distinct PPE subtypes, malignancy-associated PE (MAPE) specifically refers to PE linked to cancer-related thromboembolic events [13].

In contrast, UPE—also termed idiopathic PE—occurs without an apparent clinical risk factor. The absence of identifiable triggers may complicate diagnosis and often necessitates further investigation for occult malignancy or inherited thrombophilia [8]. UPE is associated with a higher mortality rate and generally requires prolonged anticoagulation therapy compared to PPE [10].

From a clinical standpoint, the presentation of PE varies widely, ranging from asymptomatic cases to sudden death. Common signs include tachypnea and tachycardia, while typical symptoms involve dyspnea, pleuritic chest pain, calf or thigh pain, swelling, and cough [8]. However, the non-specific nature of these symptoms can delay diagnosis, highlighting the need for early recognition to improve patient survival. Without proper treatment, PE-related mortality can reach 30%, whereas timely and effective therapy reduces it to approximately 8% [14].

Despite extensive international research [15], data on both UPE and PPE within the Israeli population remain limited. As a highly multicultural society, Israel offers a unique opportunity to explore the impact of variables such as ethnicity and sex/gender on these phenomena. This study aimed to fill this knowledge gap by analyzing the relationships between age, sex/gender, and nationality with PE occurrence, identifying high-risk populations and informing more effective diagnostic strategies. By addressing this, the research seeks to enhance patient outcomes and contribute to a more comprehensive understanding of PE within the Israeli healthcare context.

## 2. Material and Methods

### 2.1. Study Design

This retrospective observational study analyzed electronic medical records and routinely recorded administrative data of patients diagnosed with PE at Ziv Medical Center, Safed, Israel, between 2017 and 2022. The study aimed to examine the epidemiology, risk factors, and characteristics of PPE and UPE within the Israeli population. Data collection involved a comprehensive review of patient records, including demographic information (age, sex/gender, and nationality), medical history, and clinical status. Patients were categorized into two groups based on the presence or absence of identifiable clinical risk factors. Those with a history of VTE, active malignancy, heart or respiratory failure, coagulation disorders, hormone replacement therapy, oral contraceptive use, recent immobilization, surgery, or trauma within the past three months were classified as having PPE. Patients without any identifiable clinical risk factors were classified as having UPE. This classification facilitated a comparative analysis of demographic characteristics and clinical outcomes between the two groups.

Finally, ethnicity was extracted from and analyzed as a broad sociodemographic variable (Jewish vs. Arab). More granular information on sub-ethnic groups or mixed ancestry was not consistently available and therefore could not be included in the analysis.

### 2.2. Population Selection Criteria

The study cohort comprised a diverse population in terms of age, sex/gender, and nationality. Inclusion was restricted to individuals diagnosed with PE based on contemporary clinical guidelines. For the analysis of PPE, only patients with at least one established clinical risk factor were included. Conversely, those without identifiable clinical risk factors were classified as having UPE. Patients with incomplete medical records and administrative data were excluded to ensure data integrity and reliability.

### 2.3. Ethical Clearance

The study was conducted in accordance with the principles outlined in the Declaration of Helsinki, ensuring the highest standards of patient confidentiality. To protect privacy, all personal identifiers, including names and hospital identification numbers, were anonymized. The research protocol, encompassing the study design and data handling procedures, was approved by the local Helsinki Committee of the Ziv Medical Center, Safed, Israel (0025-23-Ziv and 0026-23-Ziv).

### 2.4. Statistical Analysis

Descriptive statistics were used to summarize patient characteristics, including age, gender, ethnicity, and country of birth. Continuous variables were expressed as means with standard deviations (SD) or medians, as appropriate based on the distribution assessed using the Shapiro–Wilk test. Categorical variables were presented as frequencies and percentages. Comparisons of continuous variables between groups were performed using the Mann–Whitney U test due to the non-normal distribution of the data. The chi-square test (χ^2^) was employed to assess associations between categorical variables, while Spearman’s correlation analysis was conducted to quantify associations between continuous variables. Effect sizes (ESs) for continuous variables were calculated using the biserial rank correlation. To identify predictors of PPE versus UPE, univariate analyses were initially conducted, followed by multivariable logistic regression modeling. Variables included in the logistic regression model were selected based on clinical relevance and statistical significance in univariate analysis (*p* < 0.10). The model included age, sex/gender, ethnicity, and country of birth. Odds ratios (ORs) with 95% confidence intervals (CIs) were reported. The goodness-of-fit of the model was assessed using the Hosmer-Lemeshow test. Similarly, to identify predictors of the length of stay, univariate analyses were initially conducted, followed by multivariable linear regression modeling. In-hospital mortality was analyzed using Kaplan–Meier survival curves, and differences between groups were assessed with the log-rank test. Cox proportional hazards regression was used to estimate hazard ratios (HRs) and 95% CIs for predictors of mortality. Both univariable and multivariable models were developed, with the latter adjusting for potential confounders, including age, sex/gender, ethnicity, country of birth, and PE type (PPE versus UPE). Kaplan–Meier survival curve analyses and Cox proportional hazards regression models were conducted both for all PE cases overall and stratified by PE type. The proportional hazards assumption was tested using Schoenfeld residuals. Discrimination was evaluated using the concordance index (C-statistic).

All statistical analyses were conducted using R version 4.2.3 (The R Foundation for Statistical Computing Platform, 2023). A two-tailed *p*-value of <0.05 was considered statistically significant.

## 3. Results

A total of 348 patients (mean age: 68.6 ± 17.6 years; median: 72.0) were included and analyzed. Of these, 189 (54.3%) were female and 159 (45.7%) were male. Female patients were significantly older than their male counterparts (71.7 ± 17.6 years, median: 77.0 versus 65.0 ± 16.9 years, median: 68.0; Mann–Whitney U = 10,771, *p* < 0.001). The cohort included 132 (37.9%) Arab and 216 (62.1%) Jewish patients. Jewish patients were slightly older than Arab ones (70.1 ± 16.9 years, median: 73.0 versus 66.2 ± 18.4 years, median: 72.0; Mann–Whitney U = 12,545, *p* = 0.060). Additionally, 195 patients (56.0%) were born in Israel, while 153 (44.0%) were born outside of Israel. The latter were significantly older (75.4 ± 13.4 years, median: 78.0 versus 63.3 ± 18.7 years, median: 67.0; Mann–Whitney U = 8944, *p* < 0.001).

### 3.1. Predictors of PE Type

Among the 348 cases of PE, 159 (45.7%) were unprovoked, while 189 (54.3%) were classified as provoked (Table 1). Of the PPE cases, 62 (32.8%) were attributed to surgery or trauma within the past three months, 55 (29.1%) to malignancy, 24 (12.7%) to a history of VTE, 19 (10.1%) to disabling conditions such as heart or lung failure, 16 (8.5%) to immobilization, 8 (4.2%) to hormone replacement therapy or oral contraception, and 5 (2.6%) to coagulation disorders.

In the univariate analyses, age did not differ significantly between PPE and UPE groups (Mann–Whitney U = 13,377, *p* = 0.078). The median age difference was 3.00 years, suggesting that individuals with PPE tended to be slightly younger (67.9 ± 16.5 years, median 71.0, versus 69.4 ± 18.8 years, median 74.0), though this was not statistically significant. The ES, measured using the biserial rank correlation, was 0.110, indicating a small effect. Sex/gender was not associated with PE type (χ^2^ = 0.0855, *p* = 0.770). The proportion of females was 55.0% in the PPE group and 53.5% in the UPE group, while males comprised 45.0% and 46.5%, respectively. Similarly, country of birth showed no significant association (χ^2^ = 0.000423, *p* = 0.984). The proportion of individuals born outside of Israel was nearly identical between groups (43.9% in PPE versus 44.0% in UPE), as was the proportion of those born in Israel (56.1% versus 56.0%). Ethnicity, however, was significantly associated with PE type (χ^2^ = 4.62, *p* = 0.032). Among PPE cases, 67.2% were of Jewish origin and 32.8% were Arab, compared to 56.0% and 44.0% in the UPE group, respectively (Table 1). When the distribution of PE type was examined within each ethnic group, 58.8% (127/216) of Jewish patients had PPE and 41.2% (89/216) had UPE, whereas among Arab patients, 47.0% (62/132) had PPE and 53.0% (70/132) had UPE. These findings indicate a higher relative proportion of PPE among Jewish patients and a higher relative proportion of UPE among Arab patients.

In the multivariable logistic regression analysis, age was confirmed not to be significantly associated with the likelihood of having UPE versus PPE (β = 0.00367, SE = 0.00678, *p* = 0.588; OR = 1.004 [95% CI 0.990–1.017]). Sex/gender also showed no significant predictive value, with males having an OR of 1.103 [95% CI 0.712–1.709] relative to females (β = 0.09824, SE = 0.22319, *p* = 0.660). Similarly, country of birth was not a significant factor; individuals born outside of Israel had an OR of 1.578 [95% CI 0.866–2.875] compared to those born in Israel (β = 0.45591, SE = 0.30617, *p* = 0.136). However, ethnic origin was a significant predictor, with participants of Jewish origin having significantly lower odds of UPE compared to those of Arab origin (β = −0.78221, SE = 0.29609, *p* = 0.008), with an OR of 0.457 [95% CI 0.256–0.817] (Supplementary Table S1).

Specifically concerning PPE, active malignancy was observed in 17.7% (*n* = 11) of Arab patients and 34.6% (*n* = 44) of Jewish patients. Coagulation disorders were exclusively found in the Jewish group, with a prevalence of 3.9% (*n* = 5). Disabling conditions, such as heart or respiratory failure, were more evenly distributed between the two groups, with a prevalence of 11.3% (*n* = 7) and 9.4% (*n* = 12) in Arab and Jewish patients, respectively. Hormone replacement therapy or oral contraception was reported only among Jewish patients, with a prevalence of 6.3% (*n* = 8) in this group. Immobilization was more frequent among Arab patients (14.5%, *n* = 9) compared to Jewish patients (5.5%, *n* = 7). A personal history of VTE showed similar percentages between the two groups (11.3%, *n* = 7, among Arab patients, versus 13.4%, *n* = 17, among Jewish patients). Surgery or trauma within the last three months was the most significant cause, affecting 45.2% (*n* = 28) of Arab patients and 26.8% (*n* = 34) of Jewish patients. Significant differences emerged in some causes such as the higher prevalence of malignancies and coagulation disorders among Jewish patients, while immobilization and post-traumatic outcomes were more common among Arab patients (χ^2^ = 19.0, *p* = 0.004).

### 3.2. Predictors of the Length of Stay

The average length of hospitalization was 10.7 ± 16.2 days, with a median of 6 days. In the univariate analysis, no differences according to sex/gender (Mann–Whitney U = 14,843, *p* = 0.845), ethnicity (Mann–Whitney U = 14,725, *p* = 0.836), country of birth (Mann–Whitney U = 12,947, *p* = 0.150), and PE type (Mann–Whitney U = 14,636, *p* = 0.676) could be found, while there was a statistically significant correlation between age and hospitalization length, with Spearman’s rho of 0.11, *p*-value = 0.044 (Table 1).

In the multivariable linear regression model, sex/gender showed a significant association: male patients stayed significantly longer in the hospital than females, with an estimate of 4.2333 and a standard error (SE) of 1.7650 (t = 2.398, *p* = 0.017). Similarly, types of PE were significantly associated with length of stay, with patients experiencing UPE staying less time than those with PPE (estimate = −3.9707, SE = 1.7502, t = −2.269, *p* = 0.024). Other variables, including age (estimate = 0.0239, SE = 0.0534, t = 0.446, *p* = 0.656), country of birth (non-Israel vs. Israel; estimate = −1.9459, SE = 2.3763, t = −0.819, *p* = 0.413), and ethnic origin (Jewish vs. Arab; estimate = 1.2236, SE = 2.3121, t = 0.529, *p* = 0.597), were not statistically significant predictors (Supplementary Table S2).

Concerning PPE, age was not significantly associated with length of stay (estimate = 0.0138, SE = 0.110, t = 0.1259, *p* = 0.900). In contrast, sex/gender emerged as a significant predictor: male patients had significantly longer hospital stays compared to females (estimate = 7.1246, SE = 2.870, t = 2.4821, *p* = 0.014). Neither country of birth (non-Israel vs. Israel; estimate = −0.5810, SE = 3.585, t = −0.1621, *p* = 0.871) nor ethnic origin (Jewish vs. Arab; estimate = −0.7321, SE = 3.716, t = −0.1970, *p* = 0.844) showed significant associations with hospital stay duration. Regarding underlying causes, certain clinical conditions were significantly associated with increased length of stay when compared to the reference group of patients with active malignancy. Patients with coagulation disorders had a significantly longer stay (estimate = 21.8820, SE = 9.128, t = 2.3971, *p* = 0.018), as did those with disabling conditions such as heart or respiratory failure (estimate = 24.5663, SE = 5.012, t = 4.9018, *p* < 0.001). Other causes, including hormone replacement therapy or oral contraceptive use (estimate = 2.3187, SE = 8.467, t = 0.2739, *p* = 0.785), immobilization (estimate = 6.2812, SE = 5.607, t = 1.1202, *p* = 0.264), personal history of VTE (estimate = 0.3199, SE = 4.593, t = 0.0697, *p* = 0.945), and recent surgery or trauma (estimate = 6.7699, SE = 3.621, t = 1.8696, *p* = 0.063), were not statistically significant, although recent surgery or trauma showed a trend toward longer stay.

Finally, regarding UPE, in the multivariable linear regression model, age did not demonstrate a meaningful association with length of stay, with a small and non-significant estimate of 0.0132 (SE = 0.0457, t = 0.28822, *p* = 0.774). Similarly, sex/gender (male vs. female) was not a significant predictor, as indicated by an estimate near zero (−0.0137, SE = 1.6422, t = −0.00832, *p* = 0.993), suggesting no difference in hospital stay duration between males and females. The variable country of birth (non-Israel vs. Israel) also did not reach statistical significance, although it showed a trend toward reduced stay for non-Israeli-born patients (estimate = −3.4593, SE = 2.3649, t = −1.46278, *p* = 0.146). Lastly, ethnic origin (Jewish vs. Arab) showed a positive association with hospital stay, with Jewish patients having an estimated longer stay (estimate = 4.1915, SE = 2.2614, t = 1.85352, *p* = 0.066); however, this result also did not reach conventional levels of statistical significance, though it approached borderline relevance.

### 3.3. In-Hospital Mortality Analysis

**Figure 1:**
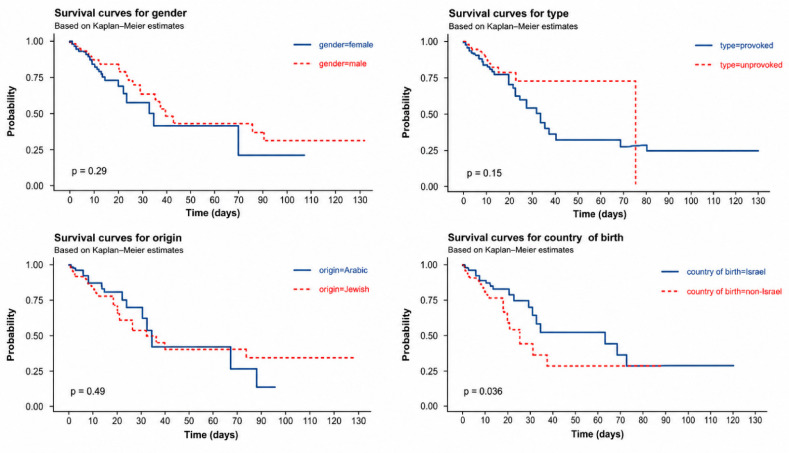
Kaplan–Meier survival curve analyses of pulmonary embolism in Israel.

The in-hospital mortality rate was 16.1%, with 56 deaths (Table 1). Overall, the Kaplan–Meier survival curve analysis (Figure 1) showed no significant impact of sex/gender (*p* = 0.29), ethnicity (*p* = 0.49), or PE type (*p* = 0.15) on survival. However, country of birth had a significant effect (*p* = 0.036), with individuals born in Israel exhibiting better survival outcomes. In the Cox regression analysis, males exhibited a non-statistically significant, lower hazard compared to females, with an HR of 0.75 ([95% CI 0.43–1.29], *p* = 0.291) in the univariable model and 0.82 ([95% CI 0.46–1.46], *p* = 0.508) in the multivariable model. Age was significantly associated with increased hazard in both models. In the univariable analysis, the HR was 1.04 ([95% CI 1.01–1.06], *p* = 0.003). This association remained statistically significant in the multivariable model, with an HR of 1.03 ([95% CI 1.00–1.06], *p* = 0.020). Individuals born outside of Israel exhibited a significantly higher hazard in the univariable model, with an HR of 1.76 ([95% CI 1.04–3.00], *p* = 0.037); however, statistical significance disappeared in the multivariable model (HR of 1.62 [95% CI 0.70–3.77], *p* = 0.263), respectively. The UPE group demonstrated a lower hazard compared to the PPE group, with an HR of 0.65 ([95% CI 0.36–1.18], *p* = 0.156) in the univariable model and 0.61 ([95% CI 0.33–1.11], *p* = 0.103) in the multivariable model. Compared to the Arab subgroup, individuals of Jewish origin had an HR of 1.22 ([95% CI 0.70–2.13], *p* = 0.481) in the univariable model and 0.83 ([95% CI 0.35–1.94], *p* = 0.664) in the multivariable model (Supplementary Figure S1, Supplementary Table S3). The concordance index for the multivariable model was 0.631 (SE of 0.046), suggesting moderate discriminative ability.

**Figure 2:**
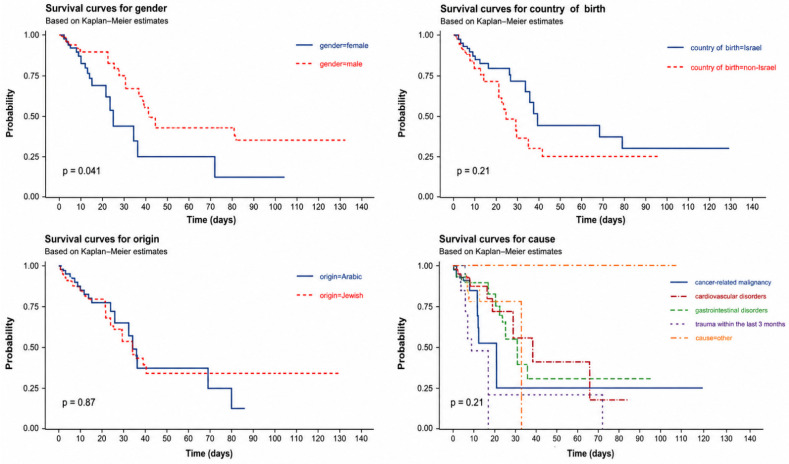
Kaplan–Meier survival curve analyses of provoked pulmonary embolism in Israel.

For PPE, the in-hospital mortality rate was 21.2%, with 40 deaths (Table 1). In the Kaplan–Meier survival curve analysis, sex/gender-specific differences (*p* = 0.041) could be found, with a lower survival among female patients. No impact of country of birth (*p* = 0.21), ethnicity (*p* = 0.87), and PE cause (*p* = 0.21) could be computed (Figure 2). In the Cox regression analysis, males had a lower hazard of mortality compared to females, with an HR of 0.51 ([95% CI 0.26–0.98], *p* = 0.045) in the univariable model and 0.54 ([95% CI 0.27–1.08], *p* = 0.080) in the multivariable model. Patients born outside of Israel demonstrated an increased hazard of mortality relative to those born in Israel, with an HR of 1.50 ([95% CI 0.80–2.80], *p* = 0.203) in the univariable model and 1.75 ([95% CI 0.65–4.76], *p* = 0.271) in the multivariable model. Jewish patients exhibited a lower mortality hazard compared to Arab patients, with an HR of 0.95 ([95% CI 0.50–1.80], *p* = 0.883) and 0.67 ([95% CI 0.25–1.77], *p* = 0.415) in the multivariable model. Age yielded an HR of 1.04 ([95% CI 1.01–1.07], *p* = 0.012) in the univariable model and 1.03 ([95% CI 1.00–1.06], *p* = 0.066) in the multivariable model (Supplementary Figure S2, Supplementary Table S4). The concordance index for the multivariable model was 0.622 (SE of 0.056), suggesting moderate discriminative ability. When stratifying by the cause of PPE, individuals who had undergone surgery or experienced trauma within the last three months exhibited a lower hazard compared to those with malignancy. In the univariable analysis, the HR was 0.59 ([95% CI 0.23–1.49], *p* = 0.262), indicating a non-significant reduction in risk. However, in the multivariable model, the association strengthened, with an HR of 0.38 ([95% CI 0.14–1.02], *p* = 0.055). For other causes of PPE, no significant associations with HRs were observed in either the univariable or multivariable models.

**Figure 3:**
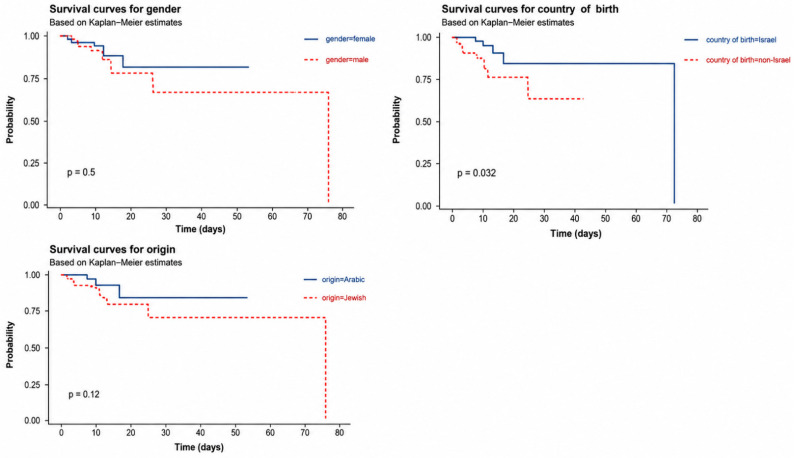
Kaplan–Meier survival curve analyses of unprovoked pulmonary embolism in Israel.

For UPE, the in-hospital mortality rate was 10.1%, with 16 deaths (Table 1). In the Kaplan–Meier survival curve analysis, no impact of sex/gender (*p* = 0.5) and ethnicity (*p* = 0.12) could be found, while an effect for country of birth (*p* = 0.032) could be detected (Figure 3). In the Cox regression, males exhibited a higher hazard of mortality compared to females, with an HR of 1.41 ([95% CI 0.51–3.90], *p* = 0.506) in the univariable model and 2.22 ([95% CI 0.76–6.50], *p* = 0.144) in the multivariable model. Patients born outside of Israel had an HR of 3.27 ([95% CI 1.04–10.28], *p* = 0.043) and 2.14 ([95% CI 0.31–14.52], *p* = 0.437) in the multivariable model, compared to those born in Israel. Jewish patients had a slightly higher hazard compared to Arab patients, with an HR of 2.63 ([95% CI 0.74–9.38], *p* = 0.135) and 1.30 ([95% CI 0.16–10.20], *p* = 0.805) in the multivariable model. Age yielded an HR of 1.04 ([95% CI 0.99–1.09], *p* = 0.103) in the univariable model and 1.04 ([95% CI 0.99–1.09], *p* = 0.150) in the multivariable model (Supplementary Figure S3, Supplementary Table S5). The concordance index for the multivariable model was 0.602 (SE of 0.026), suggesting moderate discriminative ability.

## 4. Discussion

Despite scientific achievements, PE remains a significant cause of morbidity and mortality worldwide. Previous studies in PE patients have reported significant sex/gender-specific differences [16,17,18]. Female patients tend to be older on average than male patients, as replicated in our study [16,17,18]. However, the relationship between sex/gender and mortality remains unclear. Research on the underlying factors contributing to mortality differences is limited. Understanding how symptom severity aligns with risk stratification is essential for optimizing individualized treatment and patient monitoring.

In our study, sex/gender was significantly associated with the length of hospitalization, with male patients experiencing longer hospital stays than females. When stratified by PE type, this pattern remained significant for PPE but not for UPE, where sex/gender showed no effect. No other significant associations were observed. Notably, sex/gender did not influence in-hospital mortality overall. However, when analyzed by PE type, male patients with PPE had a lower hazard of mortality compared to females, whereas the opposite trend was observed for UPE. This disparity may, in part, be attributed to female-specific risk factors for PE, such as hormonal influences, including hormone replacement therapy, oral contraceptive use, pregnancy, and the postpartum period [16,17,18].

Furthermore, previous studies [19,20] have highlighted ethnicity-specific differences in the prevalence and presentation of PE. Various racial and ethnic groups exhibit significant disparities in both PE and overall VTE incidence, although the underlying genetic, physiological, and clinical mechanisms remain largely unclear. African Americans have been shown to experience a higher incidence of VTE, particularly following provoking factors such as surgery, illness, or trauma, and are more likely to develop PE rather than DVT. In contrast, Asians and Pacific Islanders exhibit approximately 70% lower VTE prevalence across both idiopathic and provoked cases. Hispanics show a lower VTE prevalence compared to Caucasians, but a higher rate than Asians/Pacific Islanders.

In our study, Jewish patients were more likely to report PPE: specifically, 58.8% of Jewish patients had PPE compared with 47.0% of Arab patients, whereas UPE was relatively more common among Arab patients (53.0% versus 41.2%). These findings remained consistent with the statistically significant association observed in both the chi-square analysis and the multivariable logistic regression model, suggesting that ethnic differences in PE presentation may reflect variations in underlying risk factor profiles rather than differences in overall PE occurrence. Significant disparities emerged, indeed, in the distribution of specific causes: malignancies and coagulation disorders were more prevalent among Jewish patients, whereas immobilization and post-traumatic conditions were more frequently observed among Arab patients. Furthermore, individuals born outside of Israel demonstrated a significantly higher mortality risk.

These disparities highlight the urgent need for sex/gender-sensitive and population-specific diagnostic strategies and interventions that account for differences in healthcare access and socioeconomic determinants. Our study offers valuable insights into the epidemiology, risk factors, and mortality associated with PE in the Israeli population, with a particular focus on sex/gender and ethnic variations. The findings underscore the importance of developing personalized risk stratification models that integrate both demographic and clinical variables to enhance prevention and treatment strategies for PE. Future research should aim to further unravel the complex interplay between biological factors and healthcare inequalities that drive these disparities, ultimately promoting more equitable care and improved patient outcomes.

This study presents several noteworthy strengths. First, it is among the few investigations to examine PE in the Israeli population, providing valuable epidemiological and clinical insights in a multicultural setting. The use of a single-center dataset ensured consistency in diagnostic criteria and management protocols, thereby minimizing institutional variability. Moreover, the study employed rigorous statistical methods, including multivariable regression models, survival analyses, and stratified evaluations by PE type, to capture nuanced associations between demographic, clinical, and outcome variables. Notably, the analysis of sex/gender and ethnic disparities in both PPE and UPE adds a novel contribution to the literature, especially in the context of a region with significant ethnic heterogeneity. The differentiation between PPE and UPE allowed for more precise risk stratification and provided a clearer understanding of underlying etiological mechanisms.

However, several limitations should be acknowledged. The retrospective design inherently introduced the possibility of selection bias and limits causal inference. As the study was conducted in a single center, the generalizability of findings to other Israeli regions or international populations may be limited. In more detail, Ziv Medical Center is a regional tertiary hospital located in northern Israel, serving a catchment area with distinct demographic characteristics, including a higher proportion of Arab citizens compared with national averages. Consequently, the ethnic distribution of the study population reflects local population structure and referral patterns rather than the overall Israeli population. Differences in regional demographics, healthcare access, and referral pathways across Israeli hospitals may limit the generalizability of these findings. Additionally, while a wide range of clinical and demographic variables was assessed, potential confounders such as socioeconomic status, access to healthcare services, lifestyle factors, and genetic predispositions were not included and may have influenced the observed disparities. Furthermore, the reliance on electronic medical records may have led to underreporting or misclassification of comorbidities and risk factors, particularly in the case of UPE where silent or undocumented predisposing conditions may exist. Transient or situational contributors such as dehydration or short-term inflammatory conditions may not have been recorded and could therefore not be incorporated into the classification. Consequently, some events classified as unprovoked may have occurred in the presence of unmeasured transient triggers. Moreover, given the limited number of outcome events, particularly in the stratified analyses, these statistical models should be considered exploratory and hypothesis-generating, and their estimates interpreted cautiously pending validation in larger independent cohorts. Finally, a key limitation of this study is the necessarily coarse categorization of ethnicity. The binary classification of Jewish versus Arab does not capture the substantial heterogeneity within each group, nor does it account for individuals of mixed ancestry or distinct populations such as Druze or Bedouin communities. Moreover, Jewish and Arab identities encompass diverse ancestral, cultural, and geographic backgrounds. As such, ethnicity in this study should be interpreted as a pragmatic epidemiological proxy rather than a biological construct.

Future research should focus on multicenter and prospective cohort studies from different geographic and sociodemographic settings to validate these findings and enhance generalizability. Incorporating more granular data, including genetic information, socioeconomic indicators, environmental exposures, and biomarkers, would allow for a more comprehensive understanding of PE risk and outcomes. Longitudinal studies tracking post-discharge complications, recurrence, and long-term mortality could also provide critical insights into patient trajectories and inform evidence-based follow-up strategies. Finally, qualitative research exploring patient perspectives on healthcare access and diagnosis could shed light on structural barriers contributing to the disparities observed, ultimately guiding the development of more equitable and culturally sensitive healthcare interventions.

## 5. Conclusions

PE remains a major cause of morbidity and mortality, yet important demographic and clinical determinants of its presentation and outcomes remain incompletely understood. In this retrospective study of patients diagnosed with PE in northern Israel, ethnicity emerged as the only independent predictor of PE subtype, with Jewish patients being more likely to present with provoked events and Arab patients showing a relatively higher proportion of UPE. PPE was associated with longer hospitalization, whereas increasing age was the strongest predictor of in-hospital mortality. Although sex/gender, ethnicity, and PE subtype were not independently associated with mortality after adjustment for potential confounders, substantial differences in underlying risk factor profiles were observed between population groups. These findings highlight the heterogeneous nature of PE and underscore the importance of integrating demographic and clinical characteristics into risk assessment and management strategies. The observed ethnic differences in PE presentation suggest that population-specific risk factors and healthcare determinants may contribute to disease patterns and warrant further investigation. Future multicenter prospective studies incorporating socioeconomic, genetic, behavioral, and healthcare access variables are needed to better elucidate the mechanisms underlying these disparities and to support the development of more personalized, equitable, and effective approaches to PE prevention, diagnosis, and treatment.

## Figures and Tables

**Table 1:** Characteristics of the patients included.

Variable	PE (*n* = 348)	PPE (*n* = 189)	UPE (*n* = 159)
Age	68.6 ± 17.6 y; median 72	67.9 ± 16.5 y, median 71	69.4 ± 18.8 y, median 74
Sex/gender			
Female	189 (54.3%)	104 (55.0%)	85 (53.5%)
Male	159 (45.7%)	85 (45.0%)	74 (46.5%)
Ethnicity			
Arab	132 (37.9%)	62 (32.8%)	70 (44.0%)
Jewish	216 (62.1%)	127 (67.2%)	89 (56.0%)
Country of birth			
Israel	195 (56.0%)	106 (56.1%)	89 (56.0%)
Outside of Israel	153 (44.0%)	83 (43.9%)	70 (44.0%)
Hospitalization stay	10.7 ± 16.2 d; median 6	12.6 ± 20.1 d; median 6	8.55 ± 9.54 d; median 6
In-hospital mortality	16.1% (56 deaths)	21.2% (40 deaths)	10.1% (16 deaths)

Abbreviations: y, years; d, days.

## Data Availability

The database analyzed in this study is not publicly available due to patient privacy and institutional ethical restrictions. For academic and non-commercial research purposes, access can be granted upon reasonable request, subject to approval by the data governance committee and the signing of a data usage agreement. Requests should be directed to: drraymondfa@gmail.com (R.F.) and rkhamisy@yahoo.com (R.K.-F.).

## Supplementary Materials

The following supporting information can be downloaded at: https://www.mdpi.com/article/10.3390/epidemiologia7040101/s1, Figure S1: Cox regression analysis of pulmonary embolism in Israel; Figure S2: Cox regression analysis of provoked pulmonary embolism in Israel; Figure S3: Cox regression analysis of unprovoked pulmonary embolism in Israel; Table S1: Multivariable logistic regression model of pulmonary embolism type in Israel; Table S2: Multivariable linear regression model of length of stay in patients with pulmonary embolism in Israel; Table S3: Cox regression analysis of pulmonary embolism in Israel; Table S4: Cox regression analysis of provoked pulmonary embolism in Israel; Table S5: Cox regression analysis of unprovoked pulmonary embolism in Israel.

## Abbreviations

The following abbreviations are used in this manuscript:

CI	Confidence interval
C-index	Concordance index
DVT	Deep vein thrombosis
ES	Effect size
HR	Hazard ratio
KM	Kaplan–Meier
MAPE	Malignancy-associated pulmonary embolism
OR	Odds-ratio
PE	Pulmonary embolism
PPE	Provoked pulmonary embolism
R	R statistical software environment
SD	Standard deviation
SE	Standard error
UPE	Unprovoked pulmonary embolism
VTE	Venous thromboembolism
