# Supplementary material for: Epidemiology, Risk Factors, and Mortality in Unprovoked and Provoked Pulmonary Embolism—A Single-Center Retrospective Study in the Israeli Population: Gender and Ethnic Differences
Source: Epidemiologia (Basel). 2026 Jul 14;7(4):101. doi: 10.3390/epidemiologia7040101 (PMC13398013; doi:10.3390/epidemiologia7040101)

Supplementary Table S1. Multivariable logistic regression model of pulmonary embolism type in Israel.

Predictor	Estimate	Standard error	p-value	OR	95%CI lower bound	95%CI upper bound
Intercept	-0.18993	0.50647	0.708	0.827	0.306	2.232
Age	0.00367	0.00678	0.588	1.004	0.990	1.017
Male – female	0.09824	0.22319	0.660	1.103	0.712	1.709
Non-Israel – Israel	0.45591	0.30617	0.136	1.578	0.866	2.875
Jewish – Arab	-0.78221	0.29609	0.008	0.457	0.256	0.817

Supplementary Table S2. Multivariable linear regression model of length of stay in patients with pulmonary embolism in Israel.

Predictor	Estimate	Standard error	t	p-value	Standardized estimate	95%CI lower bound	95%CI upper bound
Intercept	9.0692	4.0721	2.227	0.027			
Age	0.0239	0.0534	0.446	0.656	0.0258	-0.0880	0.1397
Male – female	4.2333	1.7650	2.398	0.017	0.2607	0.0469	0.4745
Non-Israel – Israel	-1.9459	2.3763	-0.819	0.413	-0.1198	-0.4077	0.1680
Jewish – Arab	1.2236	2.3121	0.529	0.597	0.0754	-0.2047	0.3554
Unprovoked – provoked	-3.9707	1.7502	-2.269	0.024	-0.2445	-0.4565	-0.0325

Supplementary Table S3. Cox regression analysis of pulmonary embolism in Israel.

Predictor			HR (univariable)	HR (multivariable)
Age	Mean (SD)	68.6 (17.6)	1.04 (1.01-1.06, p=0.003)	1.03 (1.00-1.06, p=0.020)
Gender	female	189 (54.3)	-	-
	male	159 (45.7)	0.75 (0.43-1.29, p=0.291)	0.82 (0.46-1.46, p=0.508)
Country of birth	Israel	195 (56.0)	-	-
	non-Israel	153 (44.0)	1.76 (1.04-3.00, p=0.037)	1.62 (0.70-3.77, p=0.263)
Origin	Arab	132 (37.9)	-	-
	Jewish	216 (62.1)	1.22 (0.70-2.13, p=0.481)	0.83 (0.35-1.94, p=0.664)
Type	provoked	189 (54.3)	-	-
	unprovoked	159 (45.7)	0.65 (0.36-1.18, p=0.156)	0.61 (0.33-1.11, p=0.103)

Supplementary Table S4. Cox regression analysis of provoked pulmonary embolism in Israel.

Predictor			HR (univariable)	HR (multivariable)
Age	Mean (SD)	67.9 (16.5)	1.04 (1.01-1.07, p=0.012)	1.03 (1.00-1.06, p=0.066)
Gender	Female	104 (55.0)	-	-
	Male	85 (45.0)	0.51 (0.26-0.98, p=0.045)	0.54 (0.27-1.08, p=0.080)
Country of birth	Israel	106 (56.1)	-	-
	Non-Israel	83 (43.9)	1.50 (0.80-2.80, p=0.203)	1.75 (0.65-4.76, p=0.271)
Origin	Arab	62 (32.8)	-	-
	Jewish	127 (67.2)	0.95 (0.50-1.80, p=0.883)	0.67 (0.25-1.77, p=0.415)

Supplementary Table S5. Cox regression analysis of unprovoked pulmonary embolism in Israel.

Predictor			HR (univariable)	HR (multivariable)
Age	Mean (SD)	69.4 (18.8)	1.04 (0.99-1.09, p=0.103)	1.04 (0.99-1.09, p=0.150)
Gender	Female	85 (53.5)	-	-
	Male	74 (46.5)	1.41 (0.51-3.90, p=0.506)	2.22 (0.76-6.50, p=0.144)
Country of birth	Israel	89 (56.0)	-	-
	Non-Israel	70 (44.0)	3.27 (1.04-10.28, p=0.043)	2.14 (0.31-14.52, p=0.437)
Origin	Arab	70 (44.0)	-	-
	Jewish	89 (56.0)	2.63 (0.74-9.38, p=0.135)	1.30 (0.16-10.20, p=0.805)

Supplementary Figure S1. Cox regression analysis of pulmonary embolism in Israel.

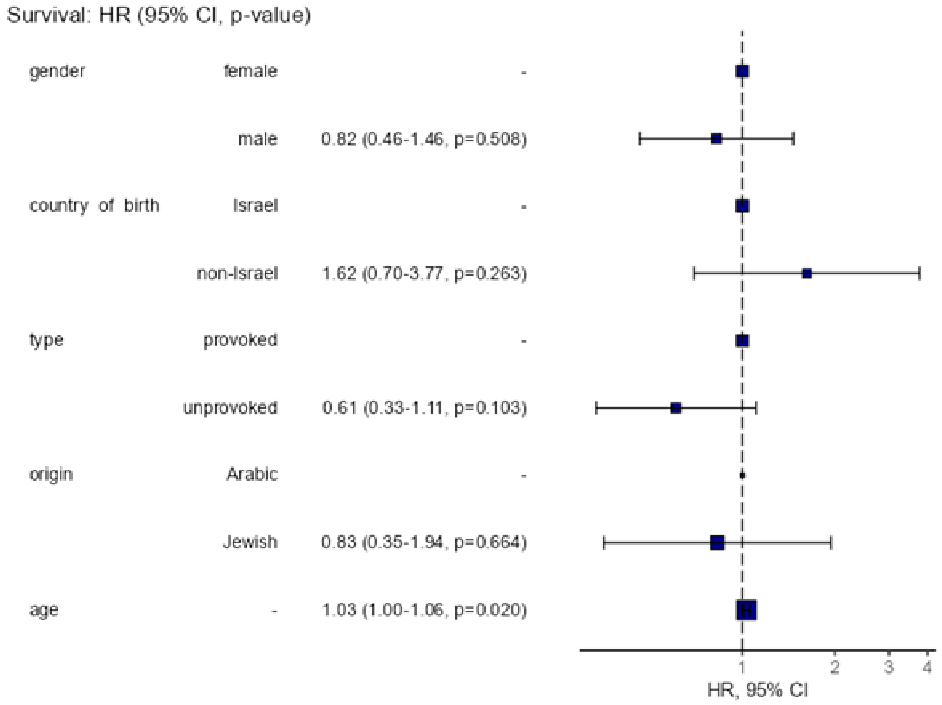

Supplementary Figure S2. Cox regression analysis of provoked pulmonary embolism in Israel.

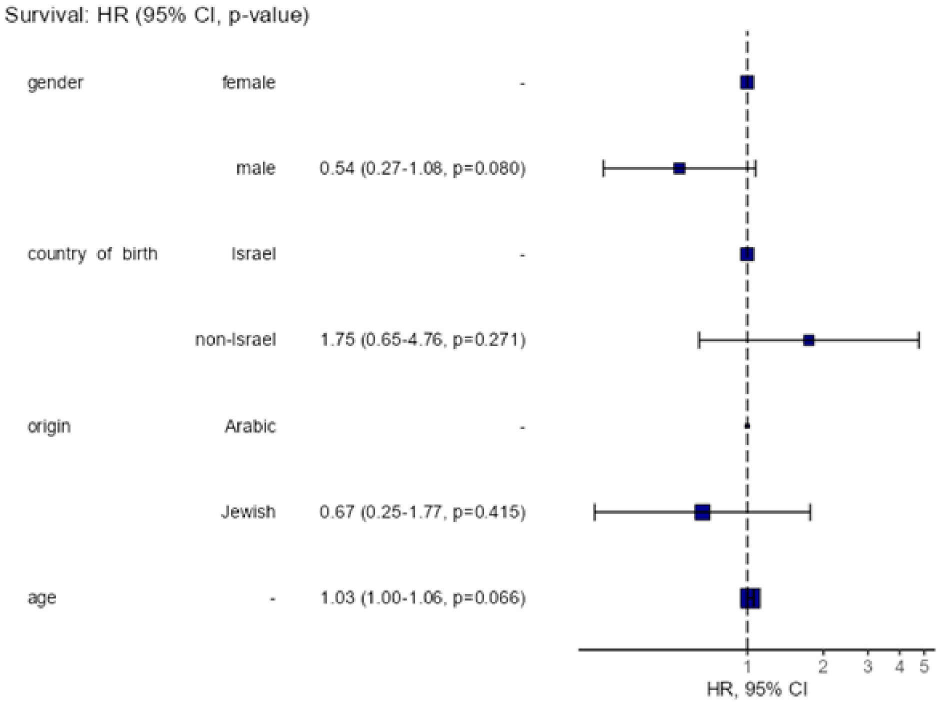

Supplementary Figure S3. Cox regression analysis of unprovoked pulmonary embolism in Israel.

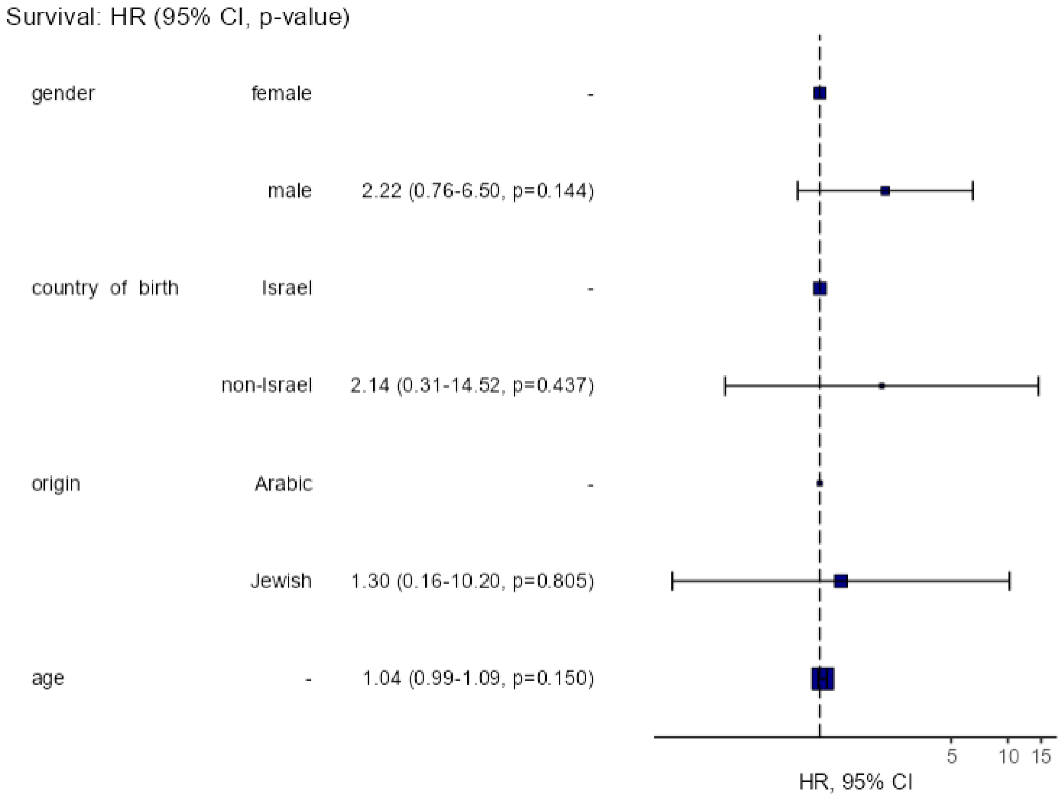

Supplement: Supplementary file 1 [file epidemiologia-07-00101-s001.zip › epidemiologia-3863481-supplementary.pdf]
